# A Neurorobotics Simulation of Autistic Behavior Induced by Unusual Sensory Precision

**DOI:** 10.1162/cpsy_a_00019

**Published:** 2018-12

**Authors:** Hayato Idei, Shingo Murata, Yiwen Chen, Yuichi Yamashita, Jun Tani, Tetsuya Ogata

**Affiliations:** Department of Intermedia Art and Science, Waseda University, Tokyo, Japan; Department of Modern Mechanical Engineering, Waseda University, Tokyo, Japan; Department of Modern Mechanical Engineering, Waseda University, Tokyo, Japan; Department of Functional Brain Research, National Center of Neurology and Psychiatry, Tokyo, Japan; Cognitive Neurorobotics Research Unit, Okinawa Institute of Science and Technology (OIST), Okinawa, Japan; Department of Intermedia Art and Science, Waseda University, Tokyo, Japan

**Keywords:** autism spectrum disorder, neurorobotics, recurrent neural network, prediction error minimization, sensory uncertainty, online adaptation

## Abstract

Recently, applying computational models developed in cognitive science to psychiatric disorders has been recognized as an essential approach for understanding cognitive mechanisms underlying psychiatric symptoms. Autism spectrum disorder is a neurodevelopmental disorder that is hypothesized to affect information processes in the brain involving the estimation of sensory precision (uncertainty), but the mechanism by which observed symptoms are generated from such abnormalities has not been thoroughly investigated. Using a humanoid robot controlled by a neural network using a precision-weighted prediction error minimization mechanism, it is suggested that both increased and decreased sensory precision could induce the behavioral rigidity characterized by resistance to change that is characteristic of autistic behavior. Specifically, decreased sensory precision caused any error signals to be disregarded, leading to invariability of the robot’s intention, while increased sensory precision caused an excessive response to error signals, leading to fluctuations and subsequent fixation of intention. The results may provide a system-level explanation of mechanisms underlying different types of behavioral rigidity in autism spectrum and other psychiatric disorders. In addition, our findings suggest that symptoms caused by decreased and increased sensory precision could be distinguishable by examining the internal experience of patients and neural activity coding prediction error signals in the biological brain.

## INTRODUCTION

Autism spectrum disorder (ASD) is a neurodevelopmental disorder that affects a broad range of cognitive functions, including perception (Simmons et al., 2009), action (Gowen & Hamilton, 2013), and social cognition (Baron Cohen, 2001). In particular, behavioral rigidity manifested as restricted, repetitive behavior and resistance to change is a core ASD symptom (American Psychiatric Association, 2013; Leekam, Prior, & Uljarevic, 2011; Poljac & Bekkering, 2012; Poljac, Hoofs, Princen, & Poljac, 2017), albeit such behavioral rigidity can be also observed in other psychiatric disorders (Lewis & Kim, 2009; Zandt, Prior, & Kyrios, 2007). Behavioral rigidity in ASD consists of various behavioral categories, such as stereotyped motor mannerisms (e.g., hand flapping) and self-injurious or compulsive behavior (Bishop et al., 2013; Lord & Jones, 2012). Although the reduced behavioral flexibility severely limits the social adaptation of patients, its cause and the underlying cognitive mechanisms remain unclear.

There have been many studies aiming to construct theories that explain the mechanisms underlying autistic symptoms (Baron Cohen, 2001; Happé & Frith, 2006; Hill, 2004), and recently the focus of these attempts has shifted to the idea of describing fundamental brain function as a set of computational processes (Redish & Gordon, 2016). In particular, theoretical explanations based on prediction error minimization frameworks, such as predictive coding (Bar, 2007; Den Ouden, Kok, & de Lange, 2012) and the free energy principle (Friston, Daunizeau, Kilner, & Kiebel, 2010), have been well investigated because they may be able to uniformly explain various ranges of autistic symptoms using a simple and neurologically plausible principle (Friston, Lawson, & Frith, 2013; Lawson, Rees, & Friston, 2014; Pellicano & Burr, 2012; Van de Cruys et al., 2014; Van de Cruys, Van der Hallen, & Wagemans, 2017; van Boxtel & Lu, 2013; van Schalkwyk, Volkmar, & Corlett, 2017). The prediction error minimization mechanism explains how we acquire knowledge and skills (learning) and how we successively infer the causes of sensory inputs and recognize environments as the process of updating a model of the world based on minimizing error between a prediction about incoming sensory inputs and actual sensory inputs. Within a scheme in which prediction error causes the brain to update its model of the world, it is crucial to estimate precision (inverse variance) of sensory information: the expected precision of certain sensory information can provide information about the reliability of the generated prediction error, which influences how much weight is given to the error when updating predictions. For example, although prediction errors for certain sensory inputs that contain information refuting the current expectation (e.g., one looks around the seabed in clear water and what seems like sand suddenly moves) should cause the brain to update its expectation (one recognizes it is not sand but flatfish), errors in sensory inputs that are very noisy (one looks around the seabed in foggy water and something moves) should not cause the update (one would think it is only a wave causing the movement). Although the estimation of such context-dependent sensory precision (prediction about whether information is informative or just noise) helps us to be flexible and adaptable in an uncertain world, deficits of it are expected to cause perceptual peculiarity and great difficulty in social contexts that are filled with situations of particularly high complexity and uncertainty (Lawson et al., 2014; Palmer, Lawson, & Hohwy, 2017; Van de Cruys et al., 2014, 2017; van Schalkwyk et al., 2017). Van de Cruys et al. (2014) suggested that inflexibly overestimated sensory precision causes autistic symptoms and inflexible behavior may be considered as an attempt to minimize prediction errors; otherwise, patients are exposed to huge error signals. Lawson et al. (2014) explained autistic behaviors as the consequences of “an imbalance of the precision ascribed to sensory evidence relative to prior beliefs.” These aberrant precision accounts of ASD in previous studies are normative and testable, but only suggestive. Specifically, there is a gap between the cognitive mechanisms described in the theories and the actual generation of the symptoms.

This kind of problem is broadly described in psychiatry, and there is a need to demonstrate actual generation of symptoms using formal computational models (Adams, Huys, & Roiser, 2015; Friston, Stephan, Montague, & Dolan, 2014; Huys, Maia, & Frank, 2016; Montague, Dolan, Friston, & Dayan, 2012; Teufel & Fletcher, 2016). Indeed, several computational simulations of psychiatric symptoms have been conducted to try to understand the processes underlying these symptoms and clarify the relationships between abnormalities at neurological and behavioral levels (Barakova & Chonnaparamutt, 2009; Brown, Adams, Parees, Edwards, & Friston, 2013; Diwadkar et al., 2008; Krichmar, 2013; O’Loughlin & Thagard, 2000; Powers, Mathys, & Corlett, 2017; Rosenberg, Patterson, & Angelaki, 2015; Yamashita & Tani, 2012). In particular, embodiment (Asada et al., 2009; Smith & Gasser, 2005) in a robot agent acting in physical environments may be useful, or even essential, for understanding the cognitive mechanisms of psychiatric disorders. That is because psychiatric disorders are characterized by behavioral and perceptual conditions observed through interaction with real environments and physical agents. In a related study, Yamashita and Tani (2012) performed a neurorobotics experiment to investigate schizophrenic cognition by utilizing a hierarchical neural network model. Their robotic experiment showed that behaviors analogous to psychiatric symptoms, such as fictive sensations and cataleptic, stereotyped behaviors, can be generated in the coupled dynamics describing the neural networks, body, and environment due to synaptic disconnections between different levels of the neural network.

In this study, we investigated the effects of increased and decreased sensory precision on adaptive behaviors by conducting experiments using a humanoid robot implemented with a version of the predictive coding model. In the experiment, a task involving adaptive interaction between the robot and a human experimenter was considered. Initially, the neural network model inside the robot learned to generate a set of sequence patterns representing different behaviors of the robot. After the learning phase, the level of estimated sensory precision was manipulated. Then, the change in the robot’s behavior in response to the alteration of the level of sensory precision was observed through experiments in which the robot was required to appropriately recognize situations determined by the experimenter. The results show that both increased and decreased sensory precision can cause seemingly similar inflexible behavioral patterns, such as inappropriate repetitive behavior and freezing; but these behaviors are the result of different processes at the network level in the two cases. Our findings may provide a system-level account for different types of behavioral rigidity observed in ASD and other psychiatric disorders and extends computational perspectives on the cognitive mechanisms underlying psychiatric symptoms.

## METHODS

### Computational Framework

**Figure 1.**
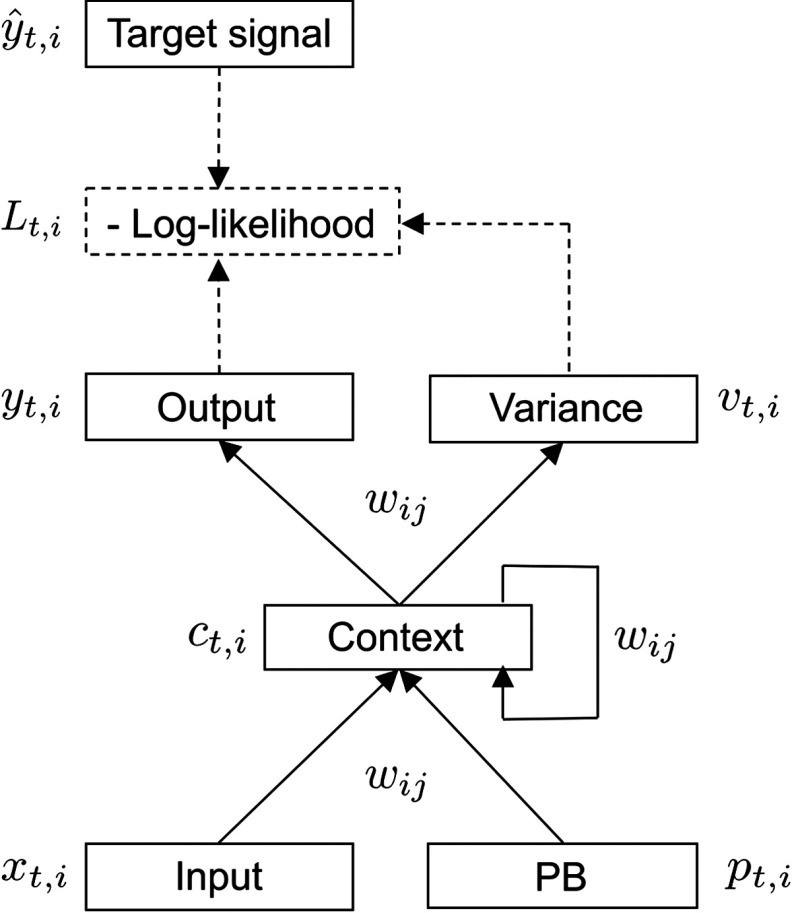
**The S-CTRNN utilized in this study.** The S-CTRNN has five groups of neural units: input, context, output, variance, and PB units. Input neural units receive current sensory inputs *x*_*t*_. Based on the inputs, PB state *p*_*t*_, and context state *c*_*t*_, the S-CTRNN generates predictions about the mean *y*_*t*_ and variance *v*_*t*_ of future inputs in the output and variance units, respectively. Parameters, such as synaptic weights *w*_*ij*_ and the internal state of PB units, are optimized by minimizing negative log-likelihood as calculated using predictions about sensory states, their variance, and actual target sensory states *ŷ*_*t*_.

We used an artificial recurrent neural network (RNN) model to investigate the effects of increased and decreased sensory precision on adaptive behaviors of a robot. An RNN is a connectionist model that can process temporal sequences thanks to recurrent connections between neural units (Elman, 1990). Owing to their capacity to learn to reproduce complex dynamic behaviors, RNNs have been used in cognitive neurorobotics studies aiming to understand human cognition (Alnajjar, Yamashita, & Tani, 2013; Marocco, Cangelosi, Fischer, & Belpaeme, 2010). Murata, Namikawa, Arie, Sugano, and Tani (2013), within the cognitive robotics scheme, proposed an RNN model with a mechanism for estimating the time-varying uncertainty of sensory information in terms of variance (inverse precision) as inspired by the free energy minimization principle proposed by Friston et al. (2010). This RNN, called a stochastic–continuous time RNN (S-CTRNN), can learn to predict not only sensory inputs but also their variances based on negative log-likelihood minimization, which is equivalent to precision-weighted prediction error minimization. Tani, Ito, and Sugita (2004) proposed an RNN with parametric bias (RNNPB) that has an online adaptation mechanism based on prediction error minimization. In this framework, parametric bias (PB) is encoded in a small group of neural units that works as a higher level neural representation of the network behavior, and the associations between specific patterns of PB activity and different temporal training patterns are self-organized through a learning process. Owing to this characteristic of PB, a robot driven by RNNPB can not only generate multiple learned behavioral patterns but also switch its behavior by adaptively modulating the PB states in response to a discrepancy between a prediction and actual sensory information. PB states thus can be regarded as the higher level “intention” of a robot Figure 1. Utilizing this model, Ito, Noda, Hoshino, and Tani (2006) demonstrated flexible switching of ball-playing behaviors by a humanoid robot in response to changes in the environment.

In the present study, an S-CTRNN with PB was adopted as the computational model for simulating aberrant sensory precision because of its capacity to learn to estimate sensory variance (precision) and adapt to different environments using a prediction error minimization mechanism. The following subsections describe in detail the mathematical procedures used for the forward dynamics and parameter optimization of the S-CTRNN with PB.

#### Forward Dynamics

The neuronal model is a conventional firing rate model. The internal state of the *i*th neural unit at time step *t*, *u*_*t*,*i*_^(*s*)^ (*t* ≥ 1), is described by

ut,i(s)=ut−1,i(s)i∈IP,1τi(∑j∈IIwijxt,j(s)+∑j∈ICwijct−1,j(s)+∑j∈IPwijpt,j(s)+bi)+(1−1τi)ut−1,i(s)i∈IC,∑j∈ICwijct,j(s)+bii∈IO,IV. (1)

Here, *I*_I_, *I*_P_, *I*_C_, *I*_O_, and *I*_V_ are index sets of the input, PB, context, output, and variance neural units, respectively; *w*_*ij*_ is the weight of the synaptic connection from the *j*th neuron to the *i*th neuron; *x*_*t*,*j*_^(*s*)^ is the *j*th input of the *s*th sequence at time step *t*; *c*_*t*,*j*_^(*s*)^ is the *j*th context state; *p*_*t*,*j*_^(*s*)^ is the *j*th PB state, *b*_*i*_ is the bias of the *i*th neuron; and *τ*_*i*_ is the time constant of the *i*th neuron. From this equation, we see that PB units can be considered to be a specific type of context unit whose time constant is infinite. The current study sets all initial values of the internal states of the context units to zero, while those of the PB units are optimized for each target sequence in the learning phase. This indicates that differences between target sequences are represented in the activity of the PB units.

The activation values of each neural unit are calculated as follows:

$$p_{t,i}^{\left(s\right)}=\tanh\left(u_{t,i}^{\left(s\right)}\right)\;\left(0\leq t\wedge i\in I_{\text{P}}\right),$$ (2)

$$c_{t,i}^{\left(s\right)}=\tanh\left(u_{t,i}^{\left(s\right)}\right)\;\left(0\leq t\wedge i\in I_{\text{C}}\right),$$ (3)

$$y_{t,i}^{\left(s\right)}=\tanh\left(u_{t,i}^{\left(s\right)}\right)\;\left(1\leq t\wedge i\in I_{\text{O}}\right),$$ (4)

$$v_{t,i}^{\left(s\right)}=\exp\left(u_{t,i}^{\left(s\right)}\right)\;\left(1\leq t\wedge i\in I_{\text{V}}\right).$$ (5)

#### Parameter Optimization

The neural network performs parameter optimization based on the gradient decent method aiming to minimize the objective function,

$$L_{t,i}^{\left(s\right)}=\frac{\ln\left(2\pi v_{t,i}^{\left(s\right)}\right)}{2}+\frac{{\left({ŷ}_{t,i}^{\left(s\right)}-y_{t,i}^{\left(s\right)}\right)}^{2}}{2v_{t,i}^{\left(s\right)}},$$ (6)

where *ŷ*_*t*,*i*_^(*s*)^ is the *i*th target value corresponding to the *s*th sequence. Minimizing this negative log-likelihood can be regarded as minimizing the precision-weighted (inverse variance–weighted) prediction error and is formally equivalent to minimizing free energy in the active inference scheme proposed by Friston et al. (2010).

In the learning phase, parameters, including synaptic weights *w*_*ij*_, biases *b*_*i*_, and the initial internal states of PB units *u*_0,*i*_^(*s*)^ (*i* ∈ *I*_P_), are updated in an offline manner. Parameter optimization is performed by minimizing the sum of the negative log-likelihood over all dimensions, time steps, and sequences as

$$L=\underset{s\in I_{\text{S}}}{\sum }\overset{T^{\left(s\right)}}{\underset{t=1}{\sum }}\underset{i\in I_{\text{O}}}{\sum }L_{t,i}^{\left(s\right)},$$ (7)

where *I*_S_ and *T*^(*s*)^, respectively, represent the index set and the length of the *s*th target sequence. The partial derivative of each parameter, (∂*L*/∂***θ***), can be found using the back-propagation-through-time (BPTT) method described in previous studies (Murata et al., 2013; Rumelhart, Hinton, & Williams, 1986).

In the adaptation phase, after learning, only the internal states of the PB units are optimized online, and other parameters are fixed. The negative log-likelihood within a short time window *W* is accumulated as

$$L=\overset{t}{\underset{t^{{}^{\prime }}=t-W+1}{\sum }}\underset{i\in I_{\text{O}}}{\sum }L_{t^{{}^{\prime }},i}^{\left(s\right)}.$$ (8)

The time window of length *W* moves along with the increment of the network time step *t*. Using the accumulated negative log-likelihood, the internal states of the PB units at time step *t* − *W* are optimized. The partial derivatives of the internal states of PB units are also calculated by the BPTT algorithm.

In both the learning and adaptation phases, parameters that are allowed to be optimized are collected as a vector ***θ***, and ***θ*** at the *n*th epoch is updated using gradient descent on the accumulated negative log-likelihood *L*:

$$\theta \left(n\right)=\theta \left(n-1\right)+\Delta \theta \left(n\right)$$ (9)

$$\Delta \theta \left(n\right)=-\alpha \frac{\partial L}{\partial \theta }+\eta \Delta \theta \left(n-1\right).$$ (10)

Here, *α* is the learning rate and *η* is a coefficient representing the momentum term. In this study, *α* and *η* are set at 0.0001 and 0.9, respectively.

### Task Setting

**Figure 2.**
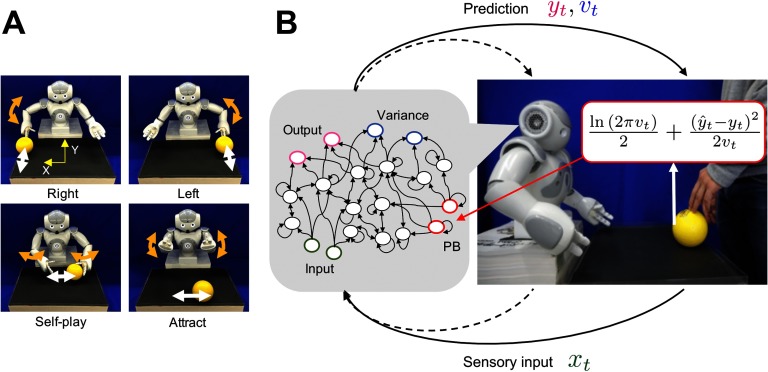
**Ball interaction tasks in the training and adaptation phases.** A) Four interactive behavioral patterns learned by a robot controled by an S-CTRNN with PB. The upper left and upper right figures show the right and left behaviors, respectively. The lower left and lower right figures show the self-play and attract behaviors. B) System overview during adaptive interaction between a robot and an experimenter. The solid lines for prediction and sensory input represent visual information about the ball position. The dotted lines represent proprioceptive information about the robot’s joint angles. The neural network generates predictions about sensory states *y*_*t*_ and their variances *v*_*t*_ based on current sensory inputs *x*_*t*_ and also recognizes situations by updating PB activity online in the direction of minimizing the negative log-likelihood calculated using the predictions and the target signal (actual sensory feedback) *ŷ*_*t*_.

To provide the robot with a task suitable for testing our hypothesis that aberrant sensory precision induces behavioral rigidity, we require a dynamical interaction setup in which the robot needs to perceive sensory information with intrinsic uncertainty and flexibly recognize situations determined by others. We chose a ball-playing scheme involving interaction between a robot and a human experimenter that was used in a previous study by Chen et al. (2016). The behavioral patterns of the robot consist of four different ball-playing behaviors (see Figure 2A). In the “right” and “left” behaviors, the robot is required to wait for the ball coming from the human subject and then return it. “Self-play” behavior consists of rolling the ball in front of itself, and the “attract” behavior is an up–down motor action with the arms while the partner engages in the “self-play” behavior of moving the ball left and right. After the S-CTRNN with PB learned to reproduce these visuo-proprioceptive temporal patterns, the behavioral performance of the robot with the trained neural network model was tested in the task of adaptive ball-playing interaction with a human subject.

### Experimental Environment

We employed a small humanoid robot NAO (Aldebaran) that has a body corresponding to only the upper half of the human body. The robot sat in front of a workbench and engaged in a ball-playing interaction with a human experimenter standing on the opposite side of the bench. The robot’s action involved only movements of the arms with 4 degrees of freedom for each arm (two shoulders and two elbows). In addition, a camera located in the robot’s mouth obtained the center of gravity coordinates for the yellow object, which was used as two-dimensional inputs for ball position. Using the minimum and maximum values of each piece of sensory information, the values of joint angles and the ball position were mapped to values ranging from −0.8 to 0.8. The size of the workbench and the diameter of the ball are approximately 45 × 5 × 30 cm and 9 cm, respectively.

### Training

Training of the neural network was conducted in an offline manner by supervised learning using target perceptual sequences recorded in advance. The target perceptual sequences were recorded while the robot repeatedly performed each ball-playing behavior, where the arm movement was generated exactly following preprogrammed trajectories instead of the ones generated by the neural network model. Each of the four behavioral patterns was obtained as a sequence of 10-dimensional vectors (8 dimensions for joint angles and 2 dimensions for ball position). For the training, three sequences were prepared for each behavioral pattern. The time lengths of the sequences were approximately 1,600 time steps for “right,” 1,900 time steps for “left,” 1,600 time steps for “self-play,” and 1,200 time steps for “attract.”

The neural network learned to reproduce these target visuo-proprioceptive sequences. The objective of the learning is to find the optimal values of the parameters (synaptic weights, biases, and internal states of PB units) minimizing negative log-likelihood, or precision-weighted prediction error. At first, each parameter was initialized with a random value, and the network produced random sequences. The parameters were updated in the direction of minimizing negative log-likelihood accumulated through the duration of the target sequences. Repeating the update process many times, the network became able to produce visuo-proprioceptive sequences with the same stochastic properties as the target sequences. In addition, the associations between a particular pattern of target sequence and specific internal states of PB units self-organized.

### Online Adaptation

After the learning process, the robot engaged in an adaptive interaction with a human experimenter by updating PB states (intention) online. In this phase, the robot’s intention was first set to a certain state corresponding to a learned behavior, and situation (ball dynamics pattern) was controlled by the experimenter. The goal of the robot was to flexibly recognize situations using visual cues. Real-time adaptation during task execution by the robot was performed based on an interaction between a top-down prediction generation process and a bottom-up parameter adaptation process. In the top-down prediction generation process, the network generated a temporal sequence corresponding to time steps from *t* − *W* + 1 to *t*, based on the sensory inputs at time step *t* − *W* + 1 and the constant PB states (intention). The visuo-proprioceptive sequence was generated by a “closed-loop” procedure, meaning that predictions about mean values of the sensory states at a certain time step were used as inputs at the next step. The initial inputs for proprioceptive states at time step *t* − *W* + 1 were taken from the generated mean predictions at *t* − *W*, and those for vision states were taken from the vision data caught by the camera at time step *t* − *W* + 1. In the bottom-up adaptation process, the negative log-likelihood at each time step within time window *W* was calculated by using the predictions about vision states, their variance, and the actual visual feedback (see Figure 2B). The PB states (intention) were updated in the direction of minimizing the accumulated negative log-likelihood. Based on the updated PB states, the temporal sequence within the time window was regenerated. After repeating these top-down and bottom-up processes for a certain number of times, the network generated its predictions for time step *t* + 1, and the predictions about proprioceptive states were sent to the robot as the target for subsequent joint positions. This procedure, where recognition and prediction in the past are reconstructed based on current sensory information, is more properly regarded as a “postdiction” process (Eagleman, 2000; Shimojo, 2014), and generated predictions for time steps from *t* − *W* + 1 to *t* are more suitably referred to as postdiction of the past rather than prediction in the literal sense.

### Parameter Setting for the Experiment

The number of input, output, and variance neural units were *N*_I_ = *N*_O_ = *N*_V_ = 10, corresponding to the dimension of the robot’s sensory states, and the number of PB units was *N*_P_ = 2. The number and time constant of the context units were *N*_C_ = 50 and *τ*_*i*_ = 4, respectively. In the learning phase, the weights of synaptic connections *w*_*ij*_ (*j* ∈ *I*_I_, *I*_C_) and biases *b*_*i*_ were initialized with random values following uniform distributions on the intervals $[-\frac{1}{N_{\text{I}}},\frac{1}{N_{\text{I}}}]\left(j\in I_{\text{I}}\right)$ and $[-\frac{1}{N_{\text{C}}},\frac{1}{N_{\text{C}}}]\left(j\in I_{\text{C}}\right)$ for weights and [−1, 1] for biases, and the internal states of PB units were initialized as 0. These parameters are updated offline 300,000 times in the learning phase. In the adaptation phase, the internal states of PB units were updated online 20 times, and the length of the time window was *W* = 10.

### Simulating Aberrant Sensory Precision

This study simulated increased and decreased sensory precision by altering estimated sensory variance (inverse precision). After the network learned to reproduce the set of behavioral patterns, the activation values of the variance units were modified as

$$v_{t,i}^{\left(s\right)}=\exp\left(u_{t,i}^{\left(s\right)}+K\right)+\epsilon \;\left(i\in I_{\text{V}}\right),$$ (11)

where *K* is a constant determining the level of the estimated variance and *ϵ* is its minimum value, set as 0.00001. *K* is set as 0 in the normal condition, while *K* is set to negative values in the decreased sensory variance conditions and to positive values in the increased sensory variance conditions (*K* ∈ {−8, −4, 0, 4, 8}).

### Analysis of Robot’s Behavior

To judge whether the robot’s behavior generated during the test phase is appropriate, the generated time series of joint angles was compared with the target (learned) time series. A simple way to compare two time series is to calculate the distance between the value at each corresponding pair of time steps within a certain time window. However, this method is not necessarily appropriate for comparing a general characteristic of time series because a phase shift will increase the distance between the series. Here this would increase the distance even when the robot generates the appropriate action. Thus this study considered histograms of time series values within a specified time window and then compared the histogram of the time series generated through the test experiment with the target time series. Because a histogram of time series values can be considered as a probability distribution, two time series can be compared by calculating the Kullback–Leibler (KL) divergence. Although the probability distribution lacks some information regarding temporal ordering, this comparative approach is suitable for our purpose because a general characteristic of a time series can be extracted. By considering the amount of the state change and calculating the KL divergence from the learned time series, the behaviors observed in the experiments could be classified into one of four types: *outwardly normal*, *freezing* (maintaining one posture), *unlearned movement* (engaging in an unlearned action), and *inappropriate learned movement* (engaging in a learned action other than the target action). These are explained in more detail in below.

To assess the robot’s behavior in the experiment, an eight-dimensional time series of joint angles was reduced to a two-dimensional time series by applying principal component analysis. To extract the probability distribution of the two-dimensional time series, the two-dimensional space [−*N*, *N*] ⋅ [−*N*, *N*] (with *N* the maximum of the absolute value of time series *S*(*t*) = {*z*_1_(*t*), *z*_2_(*t*)} across all data, where *z*_1_ and *z*_2_ represent the first and second principal components, respectively) is divided into *N*_bin_^2^ subspaces (here *N*_bin_ = 20). Then, the occurrence frequencies of states within the time series were counted. Based on the acquired probability distributions of the time series, the KL divergence between the probability distribution of the time series generated in the test experiment and the target (learned) time series was calculated. The robot’s behavior is judged as “outwardly normal” if the KL divergence is less than a threshold *ξ*, set here as half of the minimum of KL divergence between each pair of learned time series:

$$D_{\text{KL}}(p∥q)<\xi =0.5\cdot {\text{min}}_{q_{i},q_{j}\in U_{ŝ}\wedge q_{i}\neq q_{j}}D_{\text{KL}}(q_{i}∥q_{j}).$$ (12)

Here *p* is the probability distribution of the generated time series through the test experiment, *q* is the probability distribution of the target movement, and *U*_*ŝ*_ is a set of the probability distributions of each learned movement.

Atypical behaviors can be classified into one of three types of behaviors according to whether the movements were almost stopped and whether they were close to a learned movement other than the target. We call these *freezing* (if *d* < 0.02 and ∀*q* ∈ *U*_*ŝ*_, *D*_KL_ (*p*∥*q*) ≧ *ξ*), *unlearned movement* (if *d* ≧ 0.02 and ∀*q* ∈ *U*_*ŝ*_, *D*_KL_ (*p*∥*q*) ≧ *ξ*), and *inappropriate learned movement* (if *d* ≧ 0.02 and ∃*q* ∈ *U*_*ŝ*_, *D*_KL_ (*p*∥*q*) < *ξ*). In these, *d* is the amount of the state change, defined as

$$d=\frac{1}{T}\overset{T}{\underset{t=0}{\sum }}\;\;\;\underset{i\in I_{{\text{O}}_{\text{joint}}}}{\sum }|y_{i,t+1}-y_{i,t}|.$$ (13)

Here *T* is the length of the time series, *I*_O_joint__ is the index set of the joint outputs, and *y*_*i*,*t*_ is output of the *i*th output neural unit at time step *t*.

## RESULTS

### Open-Ended Ball Interaction

**Figure 3.**
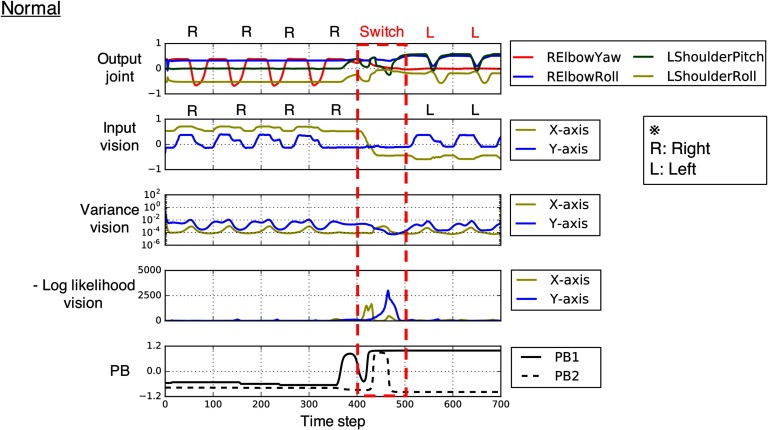
**Generated time series data from interacting with the experimenter under normal conditions.** The robot with a normal network (*K* = 0) successfully adapted to the changing situations (time steps 400–499, in red box) by flexibly switching its intention (PB state) in the direction of minimizing the increased negative log-likelihood. “Output joint” indicates predictions about selected four-dimensional joint angles. “Input vision,” “variance vision,” and “negative log-likelihood vision,” respectively, indicate the two-dimensional ball position and corresponding estimated variance and precision-weighted prediction error. The negative log-likelihood at time step *t* is the value after the postdiction process inside the error regression window between time steps *t* − *W* + 1 and *t*. PB indicates activation values of the two PB units. The joint-angle output of the time series was quantitatively assessed every 100 time steps, as described in Methods.

**Figure 4.**
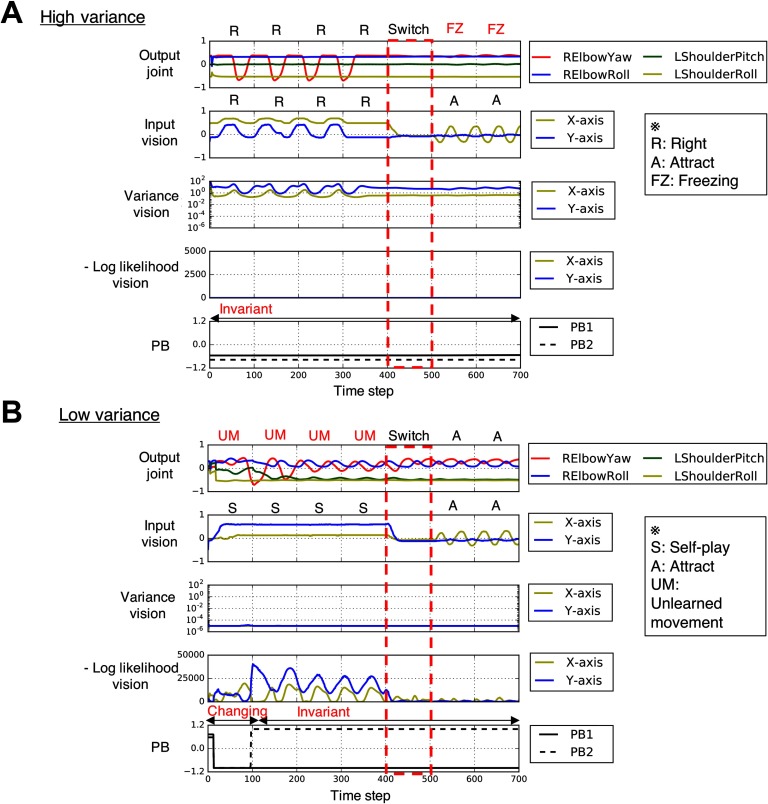
**Generated time series data from interacting with the experimenter under increased or decreased sensory variance conditions.** A) Robot’s behavior under increased sensory variance condition (*K* = 8). With increased sensory variance, the robot’s intention was invariant through the interaction with a situation change (time steps 400–499, in red box) due to highly reduced precision-weighted prediction error, leading to a freezing behavior. B) Robot’s behavior under decreased sensory variance condition (*K* = −8). With decreased sensory variance, the robot experienced huge precision-weighted prediction error signals, and its intention first quickly changed and then fixed at a certain point, leading to an unlearned repetitive movement. Note that the ranges for negative log-likelihood shown in the graphs for the high-variance condition and the low-variance condition are different. The joint-angle output of the time series was quantitatively assessed every 100 time steps, as described in Methods. Abnormal behavioral patterns, including freezing and inappropriate repetitive behavior, were observed under both increased and decreased sensory variance conditions, and these figures show representative examples.

**Figure 5.**
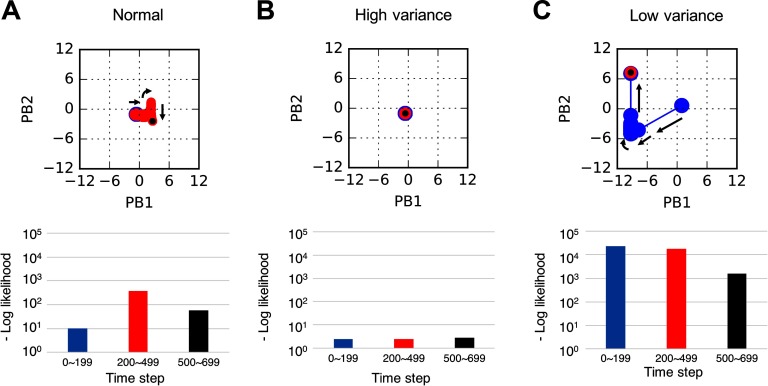
**Dynamics of internal PB states (upper figures) and error signals (bottom bar graphs) for each condition during the interactions shown in Figures 3 and 4.** Colored dots in the upper figures represent PB dynamics during different periods of time (early: time steps 0–199; middle: time steps 200–499; late: time steps 500–699). Bottom bar graphs show the corresponding mean of the negative log-likelihood per time step during each time span. A) Flexible intention switching under normal condition during the interaction shown in Figure 3. During the situation change in the middle period, generated error signals caused intention switching, and error signals were successfully reduced during interaction in the new situation in the late period. B) Deficits in intention switching for high sensory variance during the interaction shown in Figure 4A. Even when the situation changed in the middle period, PB states were almost unchanged due to the underestimated precision of prediction error. C) Large shift of network behavior for low sensory variance during the interaction shown in Figure 4B. Internal PB states first dynamically fluctuated in the early period, but after the middle period, they became almost fixed at a certain value, although generated error signals were still very large.

First, we observed the effects of increased or decreased sensory variance (inverse precision) on the robot’s behavior through an open-ended ball interaction where situations (ball dynamics patterns) were changed unpredictably by the experimenter. To assess the robot’s behaviors, the joint-angle output of the time series was quantitatively assessed every 100 time steps and classified into one of the four types of movements (see Methods). Figures 3 and 4 show some representative examples of the robot’s behaviors under each condition. Figure 5 focuses on the network-level processes during the trials shown in Figures 3 and 4.

Figure 3 shows a successful interaction between the experimenter and the robot with a normal network. The robot and the experimenter first performed a “right” interaction during time steps 0–399, then the experimenter externally changed the situation (ball dynamics pattern) to a “left” interaction during time steps 400–499 (red box in Figure 3). The unpredictable situation switch caused conflict between the robot’s intention (PB states) and the actual situation. However, the robot’s intention was soon updated in the direction of minimizing the increased negative log-likelihood (precision-weighted prediction error) (see also Figure 5A), and the robot generated behavior appropriate to the situation. This indicates that the robot with a normal network could flexibly recognize and adapt to changing environments.

On the other hand, we observed similar patterns of abnormal overt behaviors, such as freezing or inappropriate repetitive behavior by the robot, under conditions of both increased and decreased sensory variance. Figure 4A shows freezing behavior under the increased sensory variance condition. In this case, the robot first successfully performed a “right” interaction (time steps 0–399), but the robot almost stopped and maintained a single posture after the situation was switched to “attract” (time steps 500–699). Figure 4B shows an unlearned repetitive behavior under the decreased sensory variance condition. The robot’s action in this case was initially unstable (time steps 0–199) and then converged to an unlearned periodic movement (time steps 200–399), but the robot generated the appropriate movement after the situation was changed (time steps 500–699). These abnormal behaviors, such as freezing and inappropriate repetitive behavior, were observed in both the increased and decreased sensory variance conditions. The videos of the ball interactions and graphs for abnormal behaviors under increased and decreased sensory variance conditions are attached as supplementary information.

To distinguish between the mechanisms underlying the similar abnormal behaviors observed in the increased and decreased sensory variance conditions, an analysis was performed on the network-level processes and the level of precision-weighted prediction error the robot experienced (see Figures 5B and 5C). In Figure 5B (see also Figure 4A), increased sensory variance caused highly reduced precision-weighted prediction error and consequent invariability of the robot’s intention (PB states), regardless of the situation change during time steps 400–499 (red box in Figure 4A). This caused a mismatch between the robot’s intention and the situation, leading to freezing behavior. In Figure 5C (see also Figure 4B), which shows a decreased sensory variance condition, the internal PB states first quickly but incorrectly changed, possibly because the robot experienced huge precision-weighted prediction errors, which may have included errors associated with inherent noise of the ball dynamics. However, the speed of the changes slowed down when the absolute values of the internal PB states became large. After the repetitive quick state changes and a subsequent slowing down, the internal PB states were fixed at inappropriate values, even though the robot was still exposed to error signals as large as, or even larger than, it experienced before the intentional states became fixed. The fixation of intention caused a mismatch between the robot’s intention and the situation, leading to unlearned repetitive behavior. The fixation of PB states may be considered to be the result of fixing at a suboptimal local solution (suboptimal critical point) of the prediction error minimization.

The abnormal behavioral patterns characterized by resistance to change, such as freezing and inappropriate repetitive behavior, may have appeared as a result of the network dynamics converging to fixed points when there was a discrepancy between the robot’s intention and the actual situation. In addition to the behavioral abnormalities, generating appropriate behavior in a restricted situation (time steps 0–399 in Figure 4A and 500–699 in Figure 4B) was a remarkable characteristic of the observed inflexible behaviors induced by aberrant sensory variance. Thus the difficulties of the robot should not be attributed to deficits in generating organized behaviors per se but to deficits in adaptability. This behavioral rigidity characterized by resistance to change may be considered to be analogous to the characteristics of autistic behavior.

### Evaluation of Adaptability and Error Signal Level

To quantitatively evaluate the frequencies of abnormal overt behaviors described in the previous section, an additional simpler experiment was conducted. In this experiment, the situation set by the experimenter was not changed, but there was a discrepancy between the robot’s initial intention (PB states) and the situation. For example, intention of the robot was first set to the value for “left” behavior, but the experimenter rolled the ball to the right. To flexibly interact with the experimenter, the robot thus needed to switch its intention using the visual cue and generate the appropriate behavior. There were six combinations of initial PB states and ball dynamics: initial PB states were “left” or “right,” and the experimenter used one of the three other patterns of ball dynamics. Two trials were performed for each combination.

**Figure 6.**
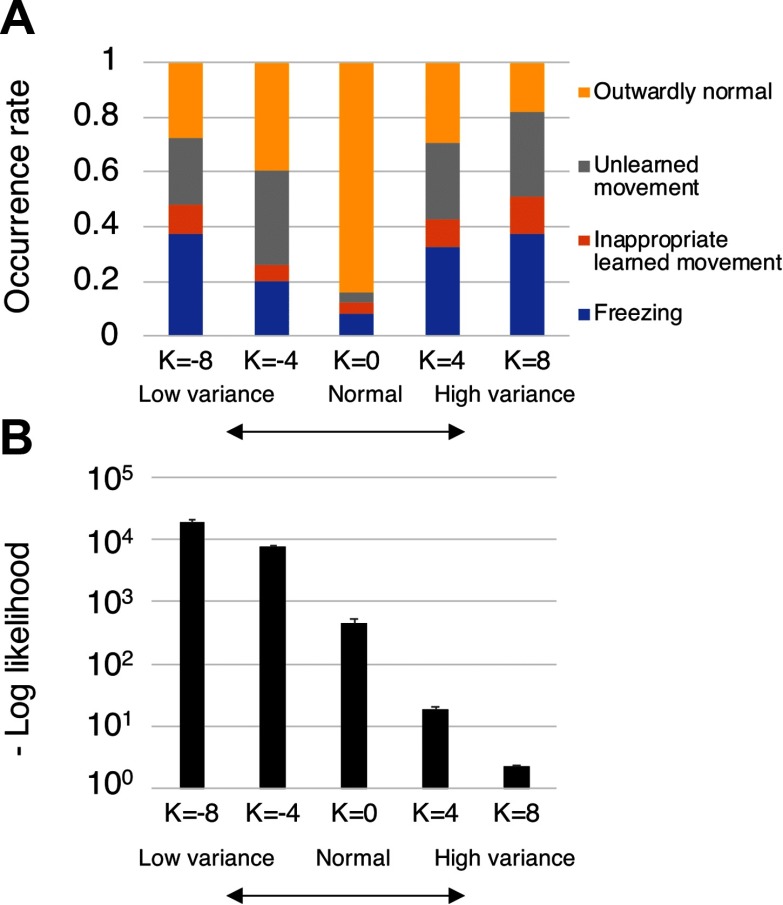
**Changes in the robot’s behavior and negative log-likelihood associated with various levels of sensory variance.** A) The occurrence rates of each behavioral trait over 120 trials for each variance level determined by a parameter *K* are shown. Behavioral traits observed at time step from 150 to 250 were assessed (see Methods). B) Negative log-likelihood per time step for each level of sensory variance is shown. Bars in the graph correspond to mean values over 120 trials for each parameter *K*. One-way repeated-measures ANOVAs indicated significant differences between the five conditions for the frequencies of the sum of the three abnormal behaviors, *F* (4, 36) = 51.0, *p* < 0.05, and levels of negative log-likelihood, *F* (4, 36) = 110.24, *p* < 0.05. Adjusting for multiple comparisons using the Holm–Bonferroni method, significant differences were found between the normal condition (*K* = 0) and other unusual variance conditions (*K* = −8, −4, 4, 8) in frequencies of abnormal behaviors, all *p* < 0.05. In addition, significant differences in levels of negative log-likelihood between all pairs were reported, all *p* < 0.05.

We evaluated the robot’s behavior in the five conditions (*K* = −8, −4, 0, 4, 8) for 10 networks trained with differently randomized initial synaptic weights. Figure 6 shows the changes in robot’s behavior and negative log-likelihood (precision-weighted prediction error) per time step associated with the levels of sensory variance. Behavioral traits observed during time steps 150–250 were assessed and divided into four overt behavioral patterns (“outwardly normal,” “freezing,” “unlearned movement,” and “inappropriate learned movement”), as described in Methods. Outwardly normal behavior basically means that the robot successfully switched its intention and generated appropriate behavior. However, it also includes behaviors for which the robot’s intention was fixed in an inappropriate state due to altered sensory variance but the robot nevertheless managed to generate appropriate behavior using only lower level network processes based on sensory inputs.

One-way repeated-measures ANOVAs and post hoc multiple comparison adjustments using the Holm–Bonferroni method (Holm, 1979) were conducted for the frequencies of the abnormal overt behaviors and the levels of negative log-likelihood. The level of statistical significance was set at *p* < 0.05. The repeated-measures ANOVAs indicated significant differences among the five conditions in the frequencies of the sum of the three abnormal movements, *F* (4, 36) = 51.0, *p* < 0.05, and the levels of negative log-likelihood, *F* (4, 36) = 110.24, *p* < 0.05. In addition, adjusting for multiple comparisons using the Holm–Bonferroni method indicated significant differences between the normal condition (*K* = 0) and the other unusual variance conditions (*K* = −8, −4, 4, 8) in the frequencies of abnormal movements (all *p* < 0.05). Significant differences in the levels of negative log-likelihood were indicated between all pairs (all *p* < 0.05). These indicate unusual sensory variance that led to unusual levels of precision-weighted prediction error, which may directly affect the perceptual processes of the robot using a prediction error minimization mechanism, thereby leading to reduction of behavioral performance.

## DISCUSSION

In this study, we tested the hypothesis that aberrant sensory precision (inverse variance) causes behavioral rigidity, a core autistic behavior. In particular, using a prediction error minimization mechanism, we investigated the effects of increased and decreased sensory variance on adaptive behaviors. We conducted experiments based on a ball interaction between a humanoid robot and a human experimenter, where the robot was required to recognize situations determined by the experimenter. Although the robot with the normal network flexibly recognized situation changes and generated appropriate interactive behaviors, both increased and decreased sensory variance (inverse precision) led to seemingly similar abnormal behaviors resulting from resistance to change, such as freezing and inappropriate repetitive behavior. However, the analysis aiming to discriminate between the mechanisms underlying similar abnormal behaviors induced by the unusual variance conditions shows there were significant differences between the network-level processes underlying the symptoms and the levels of precision-weighted prediction error signals the robot experienced. Specifically, increased sensory variance resulted in disregarding any error signals, leading to invariability of intentional state, while decreased sensory variance caused an excessive response to error signals, leading to incorrect intention change and its subsequent fixation.

Our results demonstrate that increased sensory precision (decreased sensory variance) can lead to the behavioral rigidity characteristic of ASD, supporting the system-level accounts that consider increased sensory precision as the core cognitive trait of individuals with ASD (Lawson et al., 2014; Palmer et al., 2017; Van de Cruys et al., 2014, 2017). Within a theoretical study, abnormal behavioral patterns and resistance to change in individuals with ASD were proposed as strategies to provide a reassuring sense of predictive success in a world otherwise filled with error (Van de Cruys et al., 2014). This indicates that precision-weighted prediction errors should be reduced to some extent while generating inflexible behavior. However, in our experiment, error signals could be even larger when the robot generated inflexible behavior than they were before the robot’s intention was fixed. The symptoms observed in the experiment might be understood as consequences of a suboptimal solution of prediction error minimization rather than as consequences of successfully reducing the sense of prediction error. However, the difference might be explained by the simplicity of our experimental setting. For example, in the experiment, the visual input to the robot was only from an external cause (a ball), but if visual inputs from internal causes, such as the movements of the arms, were also considered, the robot might generate characteristic behaviors aiming to minimize the total error signal from the two causes by actively changing the internal causes of vision inputs. This consideration of visual inputs from internal causes might also lead to different effects on the robot’s behaviors under increased or decreased sensory precision conditions.

Recently, problems with flexible adjustment of reactions to sensory states in response to volatile environments have been suggested to be associated with psychiatric disorders (Palmer et al., 2017; Powers et al., 2017). From previous studies, not only the unusual level of reactions but also the unusual context-sensitive adjustment of reactions, such as adaptation of precision weighting of prediction errors, might explain psychiatric symptoms. In particular, a recent empirical study indicated that autistic perception may be associated with overestimated volatility of the sensory environment, with less distinction between reactions to unexpected and expected situations (Lawson, Mathys, & Rees, 2017). In this study, sensory precision was persistently increased or decreased, indicating that influences of its context-dependent adjustment on behavioral flexibility were not considered. Future study into the effects of unusual adaptation of the precision weighting of prediction errors may facilitate understanding of finer mechanisms underlying unusual reactions to volatile environments by people with ASD. In addition, investigations will be needed of the effects of aberrant sensory precision on learning and how aberrant sensory precision can be generated through development and learning.

Our study extends attempts to understand cognitive processes underlying autistic behavior by using computational models. As a part of these attempts, Rosenberg et al. (2015) conducted neural network simulations confirming that peculiarities of vision in ASD can be induced by an altered divisive normalization. Another study associated poor motor skills in ASD with the poor goal-directed movements of a physical mobile robot induced by a deficit in temporal visuo-proprioceptive sensory integration (Barakova & Chonnaparamutt, 2009). We have confirmed that aberrant sensory precision can induce behavioral rigidity, utilizing a humanoid robot controled by a recurrent neural network model. The behavioral abnormality was observed through a real-time human–robot interaction, where the robot was required to flexibly recognize changing environments. In such uncertain and unpredictable situations, reduced cognitive flexibility of individuals with ASD has been generally reported (Leekam et al., 2011; Poljac & Bekkering, 2012). Furthermore, this study demonstrated the generation of dysfunction in intentional control (i.e., executive dysfunction) caused by aberrant sensory precision, clarifying the direct relationship between distinct proposed cognitive abnormalities in ASD (Hill, 2004; Van de Cruys et al., 2014).

Our results provide the perspective that we could consider autistic behavior as being the result of a phenomenon generally observed in natural systems. Specifically, the process leading to the qualitative shift of network behavior in the decreased sensory variance (increased sensory precision) condition may be similar to critical transitions, which are abrupt behavioral shifts observed in natural dynamical systems, including the climate, ecosystem, and cells’ signaling pathways (Lenton et al., 2009; May, 1977; Scheffer et al., 2009). Critical transitions are suggested to have characteristic early warning signals, such as the slowing down of changes in a system (critical slowing down) and back-and-forth switches between states in response to relatively large impacts (flickering), although they can also occur suddenly due to a large external impact on the system (Scheffer et al., 2009, 2012). These characteristic phenomena were observed in network behavior in the decreased sensory variance condition. This suggests that some types of behavioral rigidity and resistance to change might result from a critical transition in the hierarchical predictive control system attributed to excessive sensory prediction errors. This perspective might be implicative because pathophysiological experiments have demonstrated that dynamical features of network behavior in epileptic seizures, which relatively high numbers of individuals with ASD experience (Bolton et al., 2011), are very similar to the process of critical transition (Jiruska et al., 2010; Kramer et al., 2012).

Finally, findings from this study also provide an implication for clinical studies aiming to classify the different types of inflexible behavior observed in ASD or to understand differences between the behavioral abnormalities observed in ASD and other psychiatric disorders, such as obsessive–compulsive disorder and schizophrenia. Our results show that seemingly similar inflexible behaviors can result from different network-level processes, and also abnormalities of network-level processes may not necessarily lead to external alterations of behavior. This indicates that measurements and classifications of behavioral abnormalities based on external observation might be confusing and create difficulties in terms of understanding their etiology as broadly described in psychiatry (Redish & Gordon, 2016). However, our findings also indicate that symptoms induced by increased or decreased sensory precision were substantially different in terms of the levels of prediction error signals while generating abnormal behaviors, suggesting there might be differences in the internal experiences of individuals. Therefore measurements and classifications of both the internal experiences of patients and neural activities coding prediction error signals in the biological brain could be useful to facilitate understandings of heterogeneous behavioral rigidity in psychiatric disorders (Redish & Gordon, 2016). Future studies may be able to track parameters associated with those properties underlying disrupted adaptive behavior in animal models and humans and should compare the robot model with clinical case studies.

## AUTHOR CONTRIBUTIONS

HI, SM, YC, YY, JT, and TO conceived the research topic, designed the experiment, and wrote the paper. HI performed the experiment and analyzed the data.

## FUNDING INFORMATION

This work was supported in part by a MEXT Grant-in-Aid for Scientific Research on Innovative Areas, “Constructive Developmental Science” (24119003), JSPS Grant KAKENHI (25330301, 17K12754), and JST CREST (Grant Number: JPMJCR16E2, JPMJCR15E3), Japan.

## References

[bib1] Adams, R. A., Huys, Q. J. M., & Roiser, J. P. (2015). Computational psychiatry: Towards a mathematically informed understanding of mental illness. Journal of Neurology, Neurosurgery, and Psychiatry. 10.1136/jnnp-2015-310737 PMC4717449 26157034

[bib2] Alnajjar, F., Yamashita, Y., & Tani, J. (2013). The hierarchical and functional connectivity of higher-order cognitive mechanisms: Neurorobotic model to investigate the stability and flexibility of working memory. Frontiers in Neurorobotics, 7, 1–13. 23423881 10.3389/fnbot.2013.00002 PMC3575058

[bib3] American Psychiatric Association. (2013). Diagnostic and statistical manual of mental disorders (5th ed.). Washington, DC: Author.

[bib4] Asada, M., Hosoda, K., Kuniyoshi, Y., Ishiguro, H., Inui, T., Yoshikawa, Y., … Yoshida, C. (2009). Cognitive developmental robotics: A survey. IEEE Transactions on Autonomous Mental Development, 1(1), 12–34.

[bib5] Bar, M. (2007). The proactive brain: Using analogies and associations to generate predictions. Trends in Cognitive Sciences, 11, 280–289. 17548232 10.1016/j.tics.2007.05.005

[bib6] Barakova, E. I., & Chonnaparamutt, W. (2009). Timing sensory integration: Robot simulation of autistic behavior. IEEE Robotics and Automation Magazine, 16(3), 51–58.

[bib7] Baron Cohen, S. (2001). Theory of mind and autism: A review. Journal of the Bertrand Russell Archives, 23(169), 169–184.

[bib8] Bishop, S. L., Hus, V., Duncan, A., Huerta, M., Gotham, K., Pickles, A., … Lord, C. (2013). Subcategories of restricted and repetitive behaviors in children with ASD. Journal of Autism and Developmental Disorders, 43, 1287–1297. 23065116 10.1007/s10803-012-1671-0 PMC3579001

[bib9] Bolton, P. F., Carcani-Rathwell, I., Hutton, J., Goode, S., Howlin, P., & Rutter, M. (2011). Epilepsy in autism: Features and correlates. British Journal of Psychiatry, 198, 289–294. 10.1192/bjp.bp.109.076877 PMC3065774 21972278

[bib10] Brown, H., Adams, R. A., Parees, I., Edwards, M., & Friston, K. (2013). Active inference, sensory attenuation and illusions. Cognitive Processing, 14, 411–427. 23744445 10.1007/s10339-013-0571-3 PMC3824582

[bib11] Chen, Y., Murata, S., Arie, H., Ogata, T., Tani, J., & Sugano, S. (2016). Emergence of interactive behaviors between two robots by prediction error minimization mechanism. Paper ICDL-EpiRob 2016 presented at the 6th Joint International Conference on Development and Learning and Epigenetic Robotics, Cergy-Pontoise, France.

[bib12] Den Ouden, H. E. M., Kok, P., & de Lange, F. P. (2012). How prediction errors shape perception, attention, and motivation. Frontiers in Psychology, 3, 1–12. 23248610 10.3389/fpsyg.2012.00548 PMC3518876

[bib13] Diwadkar, V. A., Flaugher, B., Jones, T., Zalányi, L., Ujfalussy, B., Keshavan, M. S., & Érdi, P. (2008). Impaired associative learning in schizophrenia: Behavioral and computational studies. Cognitive Neurodynamics, 2, 207–219. 19003486 10.1007/s11571-008-9054-0 PMC2518754

[bib14] Eagleman, D. M. (2000). Motion integration and postdiction in visual awareness. Science, 287, 2036–2038. 10720334 10.1126/science.287.5460.2036

[bib15] Elman, J. L. (1990). Finding structure in time. Cognitive Science, 14, 179–211.

[bib16] Friston, K. J., Daunizeau, J., Kilner, J., & Kiebel, S. J. (2010). Action and behavior: A free-energy formulation. Biological Cybernetics, 102, 227–260. 20148260 10.1007/s00422-010-0364-z

[bib17] Friston, K. J., Lawson, R., & Frith, C. D. (2013). On hyperpriors and hypopriors: Comment on Pellicano and Burr. Trends in Cognitive Sciences, 17(1), 1. 23218940 10.1016/j.tics.2012.11.003

[bib18] Friston, K. J., Stephan, K. E., Montague, R., & Dolan, R. J. (2014). Computational psychiatry: The brain as a phantastic organ. The Lancet Psychiatry, 1, 148–158. 26360579 10.1016/S2215-0366(14)70275-5

[bib19] Gowen, E., & Hamilton, A. (2013). Motor abilities in autism: A review using a computational context. Journal of Autism and Developmental Disorders, 43, 323–344. 22723127 10.1007/s10803-012-1574-0

[bib20] Happé, F., & Frith, U. (2006). The weak coherence account: Detail-focused cognitive style in autism spectrum disorders. Journal of Autism and Developmental Disorders, 36(1), 5–25. 16450045 10.1007/s10803-005-0039-0

[bib21] Hill, E. L. (2004). Executive dysfunction in autism. Trends in Cognitive Sciences, 8(1), 26–32. 14697400 10.1016/j.tics.2003.11.003

[bib22] Holm, S. (1979). A simple sequentially rejective multiple test procedure. Scandinavian Journal of Statistics, 6(6), 65–70. https://www.jstor.org/stable/4615733

[bib23] Huys, Q. J. M., Maia, T. V., & Frank, M. J. (2016). Computational psychiatry as a bridge from neuroscience to clinical applications. Nature Neuroscience, 19, 404–413. 26906507 10.1038/nn.4238 PMC5443409

[bib24] Ito, M., Noda, K., Hoshino, Y., & Tani, J. (2006). Dynamic and interactive generation of object handling behaviors by a small humanoid robot using a dynamic neural network model. Neural Networks, 19, 323–337. 16618536 10.1016/j.neunet.2006.02.007

[bib25] Jiruska, P., Csicsvari, J., Powell, A. D., Fox, J. E., Chang, W.-C., Vreugdenhil, M., … Jefferys, J. G. R. (2010). High-frequency network activity, global increase in neuronal activity, and synchrony expansion precede epileptic seizures in vitro. Journal of Neuroscience, 30, 5690–5701. 20410121 10.1523/JNEUROSCI.0535-10.2010 PMC6632330

[bib26] Kramer, M. A., Truccolo, W., Eden, U. T., Lepage, K. Q., Hochberg, L. R., Eskandar, E. N., … Cash, S. S. (2012). Human seizures self-terminate across spatial scales via a critical transition. Proceedings of the National Academy of Sciences, 109, 21116–21121. 10.1073/pnas.1210047110 PMC3529091 23213262

[bib27] Krichmar, J. L. (2013). A neurorobotic platform to test the influence of neuromodulatory signaling on anxious and curious behavior. Frontiers in Neurorobotics, 7, 1–17. 23386829 10.3389/fnbot.2013.00001 PMC3564231

[bib28] Lawson, R. P., Mathys, C., & Rees, G. (2017). Adults with autism overestimate the volatility of the sensory environment. Nature Neuroscience, 20, 1293–1299. 28758996 10.1038/nn.4615 PMC5578436

[bib29] Lawson, R. P., Rees, G., & Friston, K. J. (2014). An aberrant precision account of autism. Frontiers in Human Neuroscience, 8, 302. 24860482 10.3389/fnhum.2014.00302 PMC4030191

[bib30] Leekam, S. R., Prior, M. R., & Uljarevic, M. (2011). Restricted and repetitive behaviors in autism spectrum disorders: A review of research in the last decade. Psychological Bulletin, 137, 562–593. 21574682 10.1037/a0023341

[bib31] Lenton, T. M., Myerscough, R. J., Marsh, R., Livina, V. N., Price, A. R., & Cox, S. J. (2009). Using GENIE to study a tipping point in the climate system. Philosophical Transactions of the Royal Society of London, Series A, 367, 871–884. 19087945 10.1098/rsta.2008.0171

[bib32] Lewis, M., & Kim, S. J. (2009). The pathophysiology of restricted repetitive behavior. Journal of Neurodevelopmental Disorders, 1, 114–132. 21547711 10.1007/s11689-009-9019-6 PMC3090677

[bib33] Lord, C., & Jones, R. M. (2012). Re-thinking the classification of autism spectrum disorders. Journal of Child Psychology and Psychiatry, 53, 490–509. 22486486 10.1111/j.1469-7610.2012.02547.x PMC3446247

[bib34] Marocco, D., Cangelosi, A., Fischer, K., & Belpaeme, T. (2010). Grounding action words in the sensorimotor interaction with the world: Experiments with a simulated iCub humanoid robot. Frontiers in Neurorobotics, 4, 1–15. 20725503 10.3389/fnbot.2010.00007 PMC2901088

[bib35] May, R. M. (1977). Thresholds and breakpoints in ecosystems with a multiplicity of stable states. Nature, 269, 471–477.

[bib36] Montague, P. R., Dolan, R. J., Friston, K. J., & Dayan, P. (2012). Computational psychiatry. Trends in Cognitive Sciences, 16(1), 72–80. 22177032 10.1016/j.tics.2011.11.018 PMC3556822

[bib37] Murata, S., Namikawa, J., Arie, H., Sugano, S., & Tani, J. (2013). Learning to reproduce fluctuating time series by inferring their time-dependent stochastic properties: Application in robot learning via tutoring. IEEE Transactions on Autonomous Mental Development, 5, 298–310.

[bib38] O’Loughlin, C., & Thagard, P. (2000). Autism and coherence: A computational model. Mind and Language, 15, 375–392.

[bib39] Palmer, C. J., Lawson, R. P., & Hohwy, J. (2017). Bayesian approaches to autism: Towards volatility, action, and behavior. Psychological Bulletin, 143, 521–542. 28333493 10.1037/bul0000097

[bib40] Pellicano, E., & Burr, D. (2012). When the world becomes “too real”: A Bayesian explanation of autistic perception. Trends in Cognitive Sciences, 16, 504–510. 22959875 10.1016/j.tics.2012.08.009

[bib41] Poljac, E., & Bekkering, H. (2012). A review of intentional and cognitive control in autism. Frontiers in Psychology, 3, 1–15. 23112781 10.3389/fpsyg.2012.00436 PMC3481002

[bib42] Poljac, E., Hoofs, V., Princen, M. M., & Poljac, E. (2017). Understanding behavioural rigidity in autism spectrum conditions: The role of intentional control. Journal of Autism and Developmental Disorders, 47, 714–727. 28070785 10.1007/s10803-016-3010-3

[bib43] Powers, A. R., Mathys, C., & Corlett, P. R. (2017). Pavlovian conditioning induced hallucinations result from overweighting of perceptual priors. Science, 357, 596–600. 28798131 10.1126/science.aan3458 PMC5802347

[bib44] Redish, A., & Gordon, J. (2016). Computational psychiatry: New perspectives on mental illness. Cambridge, MA: MIT Press.

[bib45] Rosenberg, A., Patterson, J. S., & Angelaki, D. E. (2015). A computational perspective on autism. Proceedings of the National Academy of Sciences of the United States of America, 112, 9158–9165. 26170299 10.1073/pnas.1510583112 PMC4522787

[bib46] Rumelhart, D. E., Hinton, G. E., & Williams, R. J. (1986). Learning representations by back-propagating errors. Nature, 323, 533–536.

[bib47] Scheffer, M., Bascompte, J., Brock, W. A., Brovkin, V., Carpenter, S. R., Dakos, V., … Sugihara, G. (2009). Early-warning signals for critical transitions. Nature, 461, 53–59. 19727193 10.1038/nature08227

[bib48] Scheffer, M., Carpenter, S. R., Lenton, T. M., Bascompte, J., Brock, W., Dakos, V., … Vandermeer, J. (2012). Anticipating critical transitions. Science, 338, 344–348. 23087241 10.1126/science.1225244

[bib49] Shimojo, S. (2014). Postdiction: Its implications on visual awareness, hindsight, and sense of agency. Frontiers in Psychology, 5, 1–19. 24744739 10.3389/fpsyg.2014.00196 PMC3978293

[bib50] Simmons, D. R., Robertson, A. E., McKay, L. S., Toal, E., McAleer, P., & Pollick, F. E. (2009). Vision in autism spectrum disorders. Vision Research, 49, 2705–2739. 19682485 10.1016/j.visres.2009.08.005

[bib51] Smith, L., & Gasser, M. (2005). The development of embodied cognition: Six lessons from babies. Artificial Life, 11(1–2), 13–29. 15811218 10.1162/1064546053278973

[bib52] Tani, J., Ito, M., & Sugita, Y. (2004). Self-organization of distributedly represented multiple behavior schemata in a mirror system: Reviews of robot experiments using RNNPB. Neural Networks, 17, 1273–1289. 15555866 10.1016/j.neunet.2004.05.007

[bib53] Teufel, C., & Fletcher, P. C. (2016). The promises and pitfalls of applying computational models to neurological and psychiatric disorders. Brain, 139, 2600–2608. 27543973 10.1093/brain/aww209 PMC5035823

[bib54] Van de Cruys, S., Evers, K., Van der Hallen, R., Van Eylen, L., Boets, B., De-Wit, L., & Wagemans, J. (2014). Precise minds in uncertain worlds: Predictive coding in autism. Psychological Review, 121, 649–675. 25347312 10.1037/a0037665

[bib55] Van de Cruys, S., Van der Hallen, R., & Wagemans, J. (2017). Disentangling signal and noise in autism spectrum disorder. Brain and Cognition, 112, 78–83. 27651171 10.1016/j.bandc.2016.08.004

[bib56] van Boxtel, J. J. A., & Lu, H. (2013). A predictive coding perspective on autism spectrum disorders. Frontiers in Psychology, 4, 1–3. 23372559 10.3389/fpsyg.2013.00019 PMC3556598

[bib57] van Schalkwyk, G. I., Volkmar, F. R., & Corlett, P. R. (2017). A predictive coding account of psychotic symptoms in autism spectrum disorder. Journal of Autism and Developmental Disorders, 47, 1323–1340. 28185044 10.1007/s10803-017-3065-9

[bib58] Yamashita, Y., & Tani, J. (2012). Spontaneous prediction error generation in schizophrenia. PLoS One, 7(5). 10.1371/journal.pone.0037843 PMC3364276 22666398

[bib59] Zandt, F., Prior, M., & Kyrios, M. (2007). Repetitive behaviour in children with high functioning autism and obsessive compulsive disorder. Journal of Autism and Developmental Disorders, 37, 251–259. 16865546 10.1007/s10803-006-0158-2

